# Genetic analyses of oculocutaneous albinism types 1 and 2 with four novel mutations

**DOI:** 10.1186/s12881-019-0842-7

**Published:** 2019-06-13

**Authors:** Qi Yang, Sheng Yi, Mengting Li, Bobo Xie, Jinsi Luo, Jin Wang, Xiuliang Rong, Qinle Zhang, Zailong Qin, Limei Hang, Shihan Feng, Xin Fan

**Affiliations:** Department of Genetic and Metabolic Central Laboratory, Guangxi Maternal and Child Health Hospital, No.59, Xiangzhu Road, Nanning, China

**Keywords:** Oculocutaneous albinism, TYR, OCA2, Mutation, Chinese

## Abstract

**Background:**

Oculocutaneous albinism (OCA) is a human autosomal-recessive hypopigmentation disorder with hypopigmentation in the skin, hair, and eyes. OCA1 and OCA2 are caused by mutations of the *TYR* and *OCA2* genes, respectively, which are responsible for most oculocutaneous albinism. However, the incidence of oculocutaneous albinism patients in Guangxi remains unclear.

**Methods:**

To evaluate the molecular basis of oculocutaneous albinism in thirty-six patients in Guangxi, China. Peripheral venous blood samples were collected from these unrelated patients. The *TYR* and *OCA2* genes of all individuals were analyzed by direct DNA sequencing and the sequences compared with are reference database and bioinformatics analysis.

**Results:**

Among the 36 OCA patients, 8(22.2%) were found mutations on *TYR* gene*, 28* (77.8%) on *OCA2*. And we identified Twenty-seven different *TYR* and *OCA2* mutations in these patients, including one novel *TYR* framshift mutation c.561_562insTTATTATGTGTCAAATTATCCCCCA, three novel OCA2 mutations: one nonsense mutation c.2195C > G(p.S732X), one deletation mutation(c.1139-1141delTGG), one missense mutations c.2495A > C(p.H832P). The population screening and the bioinformatic analysis to determined the effects of the mutations, which revealed these four novel mutations were pathogenic.

**Conclusions:**

This study expands the mutation spectrum of oculocutaneous albinism. Four novel mutational alleles c.1139-1141delTGG, c.1832 T > C and c.2195C > G and of the *OCA2* gene and c.561_562insTTATTATGTGTCAAATTATCCCCCA of *TYR* were associated with OCA. The genotype–phenotype correlations suggest that molecular diagnosis is more accurate and important in OCA.

**Electronic supplementary material:**

The online version of this article (10.1186/s12881-019-0842-7) contains supplementary material, which is available to authorized users.

## Background

Albinism is a group of hereditary disorders caused by a deficit in production of the pigment melanin. It can be classified as oculocutanous albinism (OCA) and ocular albinism (OA) [1]. The most common and visible type of albinism is oculocutaneous albinism, which is a group of autosomal recessive disorders with a reduction or complete absence of melanin in the skin, hair, and eyes and is often associated with ocular changes including photophobia, decreased visual acuity and nystagmus [2]. OCA is subdivided into 7 subtypes (OCA 1–7) based on genes as follows: TYR (OCA1), OCA2 (OCA2), TYRP1 (OCA3), SLC45A2(OCA4), SLC24A5 (OCA6), LRMDA (OCA7) and OCA5 located on chromosome 4q24 and the subtypes can only be accurately diagnosed by genetic gene [3]. OCA affects one in 17,000 individuals worldwide [4]. The prevalence of OCA subtypes differs among ethnic groups. OCA1 has been reported to be the most subtype in Caucasians and accounts for approximately 50% of cases worldwide [5, 6]. OCA2 is the most common form of albinism worldwide; in which the prevalence is estimated as 1/30000 due to Caucasians [5], and in the African-American population, in which the prevalence is estimated to be 1:10,000 [6]. OCA3,or rufous OCA (ROCA), it has been reported to affect 1:8500 individuals in Africa, but is virtually unseen in Caucasians and Asiatic populations. OCA3,or rufous OCA (ROCA), it has been reported to affect 1:8500 individuals in Africa, but is virtually unseen in Caucasians and Asiatic populations [7]. OCA4 were reported to explain the disease in approximately 18% of Japanese patients, whereas it is very rare in in Korean, Chinese,Caucasians populations [7, 8]. In Han Chinese, the prevalence of OCA is about 1:18000, and OCA1 is the most subtype [9].

OCA1, is caused by mutations of *TYR* on chromosome 11q14 and it exhibits the most severe phenotype [10]. More than 300 mutations in *TYR* have been identified in individuals with OCA1 (MIM 203100) phenotype [11]. There are two subtypes of OCA1: OCA1A and OCA1B. OCA1A is caused by a mutation causing a complete lack of tyrosinase activity, which presents with milky skin and white hair throughout life. Whereas type 1B (OCA1B) is caused by the mutations causing reduced activity of tyrosinase, and the white/light yellow hair and white skin of individuals with OCA1B can darken overtime [4, 12]. Oculocutaneous albinism type II (OCA2-MIM 203200), an autosomal recessive disorder in which the biosynthesis of melanin pigment is reduced in skin, hair, and eyes, which has been described in all major ethnic groups [13]. OCA2 is caused by mutations of *OCA2* (previouslycalled P gene), which is located on chromosome 15q11.2-q12 and consists of 24 exons (23 coding). More than 140 mutations have been identified in its gene body (HGMD Professional http://www.hgmd.cf.ac.uk/ac/all.php). Although the function of *OCA2* is not precisely characterized, the p protein consists of 12 transmembranes panning regions and is an integral component of themelanosomal membrane [13]. But it could be involved in the transport of tyrosine, the precursor to melanin synthesis, within the melanocyte. Regulates the pH of melanosome and the melanosome maturation [14–16].

As mutations in the *TYR* and *OCA2* genes account for the majority of OCA cases, we have analyzed and examined the *TYR* and *OCA2* genes in thirty-six patients with oculocutaneous albinism in Guangxi Zhuang Autonomous Region of China in the present study to identify the causative mutations for each of them.

## Methods

### Patients

August 2017 to May 2019, thirty-six unrelated OCA patients (33 minors and 3 adults) and their parents and 300 unaffected subjects were recruited in our study. All the patients were from the provinces of Guangxi, China. The families were referred to the Guangxi Maternal and Child Health Hospital for advice about diagnosis or highly suspected in patients with non-syndromic ocular albinism and Genetic testing demands of the patient. We followed the criteria for the differentiation of OCA1A, OCA1B and OCA2 as described [16]. Among the 36 OCA patients, sixteen were clinically diagnosed as OCA1A or OCA1B, twenty were diagnosed as OCA2 (Table 1). In all the 36 OCA patients, varying colors of the skin and hair, and abnormal ophthalmological findings including photophobia, nystagmus and reduced visual acuity were observed. The 300 normal controls matched by gender and ethnic origin were selected and recruited from healthy individuals from the Center of Genetic Metabolism Genetic Metabolism at Guangxi Maternaland Child Health Hospital. All participants agreed to carry out a genetic analysis and singed written informed consent for the study approved by the Genetic and Metabolic Central Laboratory of Guangxi Zhuang Autonomous Region Women and Children Care Hospital (Nanning, China).

**Table 1:** Clinical features and mutations for thirty-one Chinese patients of Oculocutaneous albinism

Patient NO	Gender	Age range (Years; A: 0–16, B: > 16)	Clinical diagnosis	Molecular diagnosis	Mutations	
					Paternal	Maternal
TYR						
1	F	A	OCA1A	OCA1A	c.896G > A (p.R299H)	c.896G > A (p.R299H)
2	F	A	OCA1B	OCA1A	c.1199G > T(p.W400 L)	c.230G > A(p.R77Q)
3	M	B	OCA1A	OCA1A	c.896G > A (p.R299H)	c.929dupC(p.R311Kfs)
4	F	A	OCA1A	OCA1B	c.996G > A (p.M332I)	c.1265G > A (p.R422Q)
5	F	A	OCA1A	OCA1A	c.230G > A (p.R77Q)	c.230G > A (p.R77Q)
6	F	A	OCA1A	OCA1A	c.929dupC(p.R311Kfs)	c.929dupC(p.R311Kfs)
7	M	A	OCA1A	OCA1A	c.346C > T (p.R116X)	c.346C > T (p.R116X)
8	M	A	OCA1A	OCA1A	c.561_562insTTATTATGTGTCAAATTATCCCCCA(G190Cfs*12)	c.561_562insTTATTATGTGTCAAATTATCCCCCA(G190Cfs*12)
OCA2						
9	F	A	OCA1A	OCA2	c.593C > T(p.P198L)	c.593C > T(p.P198L)
10	F	A	OCA2	OCA2	c.808-3C > G	C.808-3C > G
11	F	A	OCA2	OCA2	c.1327G > A (p.V443I)	c.1423A > C (p.T475P)
12	F	A	OCA1A	OCA2	c.1327G > A (p.V443I)	c.1363A > G (p.R455G)
13	M	A	OCA2	OCA2	c.1139-1141delTGG	c.1327G > A (p.V443I)
14	M	A	OCA1A	OCA2	c.2195C > G (p.S732X)	c.593C > T (p.P198L)
15	F	B	OCA2	OCA2	c.1560–1562 delCCT	c.2495A > C(p.H832P)
16	F	A	OCA1B	OCA2	c.1441G > A (p.A481T)	c.1182 + 1G > A
17	M	A	OCA2	OCA2	c.808-3C > G	c.2363 T > C (p.S788 L)
18	F	A	OCA2	OCA2	c.1832 T > C (p.L611P)	c.808-3C > G
19	F	A	OCA1A	OCA2	c.1832 T > C (p.L611P)	c.2359G > A (p.A787T)
20	F	A	OCA1A	OCA2	c.1832 T > C (p.L611P)	c.1349C > T (p.T450 M)
21	F	A	OCA1A	OCA2	c.1832 T > C (p.L611P)	c.1349C > T (p.T450 M)
22	F	A	OCA1B	OCA2	c.1832 T > C (p.L611P)	c.1832 T > C (p.L611P)
23	M	A	OCA2	OCA2	c.1832 T > C (p.L611P)	c.1832 T > C (p.L611P)
24	M	A	OCA2	OCA2	c.1832 T > C (p.L611P)	c.1832 T > C (p.L611P)
25	F	A	OCA2	OCA2	c.1832 T > C (p.L611P)	c.2195C > G(p.S732X)
26	F	A	OCA2	OCA2	c.1327G > A(p.V443I)	c.1832 T > C (p.L611P)
27	F	A	OCA2	OCA2	c.808-3C > G	c.1832 T > C (p.L611P)
28	M	A	OCA2	OCA2	c.808-3C > G	c.1832 T > C (p.L611P)
29	M	A	OCA2	OCA2	c.1182 + 1G > A	c.1832 T > C (p.L611P)
30	M	B	OCA2	OCA2	c.1001C > T(p.A334V)	c.1832 T > C (p.L611P)
31	F	A	OCA2	OCA2	c.808-3C > G	c.632C > T(p.P211L)
32	M	A	OCA2	OCA2	c.632C > T (p.P211L)	c.808-3C > G
33	M	A	OCA1B	OCA2	c.1714C > T(p.R572C)	c.2180 T > C(p.L727P)
34	M	A	OCA2	OCA2	c.2180 T > C(p.L727P)	c.1327G > A(p.V443I)
35	M	A	OCA2	OCA2	c.1349C > T(p.T450 M)	c.1832 T > C(p.L611P)
36	F	A	OCA2	OCA2	c.1363A > G(p.R455G)	c.808-3C > G

### Mutation screening

Peripheral blood samples were collected from thirty-six unrelated patients and 300 unaffected subjects, and genomic DNA was extracted from peripheral blood of the trios using Lab-Aid DNA kit (Zeesan Biotech Co, Ltd., China), DNA concentration was determined with NanoDrop ND-2000 spectrophotometer and soft-ware (NanoDrop Technologies, Berlin, Germany). Polymerase chain reaction (PCR) primers were designed using Primer3 (http://bioinfo.ut.ee/primer3-0.4.0/) to cover five coding exons of the *TYR* gene and 2–24 exons of the *OCA2* gene (Tables 2 and 3). Appropriate annealing temperatures were selected for PCR. PCR products were analyzed with agarose gel electrophoresis and used for Sanger sequencing. When a potential novel mutation was considered after careful check with the ClinVar (http://www.ncbi.nlm.nih.gov/), HGMD (http://www.hgmd.cf.ac.uk/ac/), HPSD (http://liweilab.genetics.ac.cn/HPSD/), Albinism Datebase, (http://www.ifpcs.org/albinism/oca2mut.html) and the SNP (http://www.ncbi.nlm.nih.gov/SNP/) databases, direct sequencing of the amplified PCR products from the same region of the 300 unaffected subjects was applied to exclude the possibility of polymorphism.

**Table 2:** PCR primers and conditions used for mutation analysis of the tyrosinase gene

Exon	Sequence(5′-3′)			
	Forword	Reverse	Product size(bp)	Annealing temperature(°C)
1	TGCTGGAGGTGGGAGTGGTATT	AAATACAAGATACATTGAGAGT	864	56
2	ATTTCTGCCTTCTCCTAC	TAGGACTTTGGATAAGAGACTGTAA	354	56
3	CCAGAATGTAAAGAGTCTCAATACGGAA	TCACAGACAATAGACTACCATAACT	421	56
4	TATGTACCACTTAACTGTG	ACTGCTCTTCACATGGTTGC	2269	62
5	GCCTTCAAACCCAGGTGTCTAC	CACATACAAATAACAGTTCCTC	455	56

**Table 3:** PCR primers and conditions used for mutation analysis of the *OCA2* gene

Exon	Sequence(5′-3′)			
	Forword	Reverse	Product size(bp)	Annealing temperature(°C)
2	ATGCTGGAACTCTGGGACC	GGAACGATGCTCATGGAAAC	438	60
3	GGTCTTTCTTATGGTGTCTTC	TCTCAAGTTCTCCAGCATAC	359	60
4	CAGGGTTGATTCGGTGCCAT	CTTCTTCACGCTGCTGGTTTG	390	60
5	AAGTGTCTGAGTCTGGGCAAC	GGCTGAACAGGGAAGTGGTAAG	442	60
6	CAGTAGCCCCATCATCACATC	CCTTCAGCAGCAGTCACAAC	269	60
7	AACGCATTTCTTCACACACTG	CCCATCAAATCCATTCAAGAG	354	60
8	GTTGGGATTACAGGCGTGAG	TGCTGACCTGGTGCTGTGTG	537	60
9	GCCTGTGCTCACTGCTCTTC	TTCCTGTATGGTTCCCTTTCT	476	60
10	TGTATGTGTCTGTGGGGTGTC	CGAAAGCCTGAATCCTGGAAC	323	60
11	GGCAAGTGGATGGTGAGATTTC	CCATAGCCCCATTCCATTC	375	60
12	ATGTGGTGGCTTTCAGAG	TGAGTACCCTTTTCCTTGAC	405	60
13	TGTTAGTTTGGCTCCCTGTTC	CCTATGTCTTCCACCTCCTG	469	60
14	AGGGTTTGGTGGCTGGAGG	GTGGAGGTGTGCGTTTACTGG	421	62
15	CACGCCATTCTCCTGCCTC	CATCCAGCAACCCATCAAC	468	60
16	ATGTCGGCTTTGTCGTCTG	CTCGGCTGTGTACCCCCTG	520	60
17	CCAGCCAACAAATGAAGCC	TCCCCATCCACTCACACAC	379	60
18	TGTCGTGATTCCAGTTGCGT	CCCTCCATCTCAGCCCTCTC	332	60
19	TCTTGATTACAGTGTTGGTT	CTTCATTGTTTTCCACTTAG	442	60
20	CTGCTGTTGGACTTTTTC	ATTTACTGAGGCTGGTGT	538	60
21	TCGTGATGGGTAAGAGGAAGG	AAGCAAGCAAGCAAGCACAG	528	60
22	TCAGGACCAGGAGGACCAGTTG	CAGAGAATGGGAAGGAACGGAG	507	60
23	TGCGTGTGTGTGTGTTTCC	AATCTCCCCTACACCACAGTC	389	60
24	ATAGATGAACAAACAGAGGCT	GAAAGGACACACAGAGGAGG	566	60

### In silico analysis

Four softwares were used to analyze the functional effects of novel variants, including SIFT (http://sift.jcvi.org/), PolyPhen2 (http://genetics.bwh.harvard.edu/pph2/), Mutation Taster (www.mutationtaster.org). Multiple amino acid sequence align?ment from different species was generated by HomoloGene (https://www.ncbi.nlm.nih.gov/homologene). Classification of variants according to the ACMG standards and guidelines [17].

## Results

### Mutation identification and analysis

**Fig. 1:**
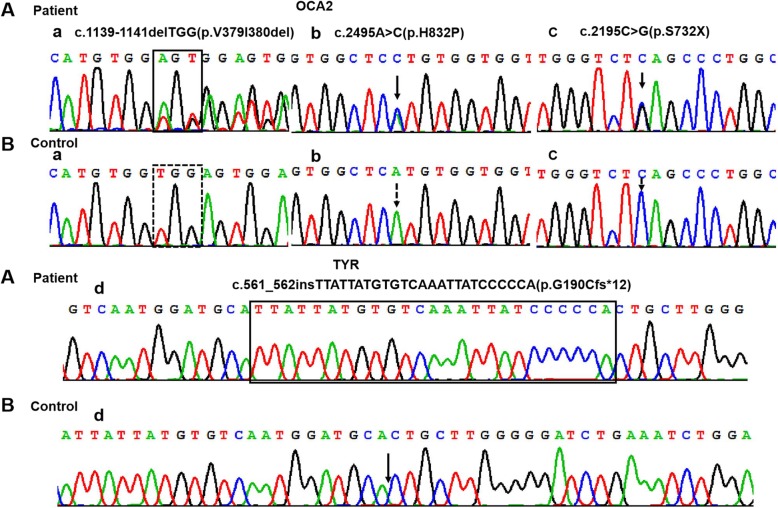
DNA sequencing result from OCA2 gene (a**-**c) or TYR (d) gene. Chromatograms demonstrating the nucleotide changes detected. Black arrows or boxes indiae the c.1139-1141delTGG, c.2495A > C(p.H832P), c.2195C > G(p.S732X) and c.561_562insTTATTATGTGTCAAATTATCCCCCA(p.G190Cfs*12) mutations. The nucleotide sequence of(**a**:a-f) the patients and (**b**:a-f) the representative normal subjects

Sanger sequencing analysis of the relevant PCR fragments in exons of the *TYR* and *OCA2* genes revealed twenty-five different variants in thirty-one unrelated individuals. The variants included ninteen known mutations: nine *TYR* mutations and thirteen OCA2 mutations (Table 1); one novel *TYR* framshift mutation c.561_562insTTATTATGTGTCAAATTATCCCCCA(G190Cfs*12), three novel OCA2 mutations including one nonsense mutation c.2195C > G(p.S732X), one deletation mutations (c.1139-1141delTGG), one missense mutations c.2495A > C(p.H832P), all novel variants were not detected in our 300 control individuals (Fig. 1). Among these 36 OCA patients, 26 patients were identified of compound heterozygous mutations, and other 10 patients were were identified of homozygous mutations (Additional file 1) gene, changes also seen in the father and mother.). The frequency percentage of the detected OCA2 mutations are shown in Table 4. Of these, the c.1832 T > C(p.L611P) mutation was most frequent (32.1%), followed by the c.808-3C > G mutation (16.10%), c.1327G > A(p.V443I) (8.90%), c.1349C > T(p.T450 M) (5.40%) and c.593C > T(p.P198L) (5.40%); the other mutations were observed only once or twice.

**Table 4:** Allelic frequencies of the OCA2 gene in 26 Chinese OCA2 patients

Nucleotide change	Amino acid change	Status (Number of the patients)	Frequency percentage(%)
C.808-3C > G	splice site	Homo (1), Hetero (6)	15.4
c.1327G > A	p.V443I	Hetero (5)	9.6
c.1423A > C	p.T475P	Hetero (1)	1.9
c.1363A > G (p.R455G)	p.R455G	Hetero (1)	1.9
c.1139-1141delTGG	Other site	Hetero (1)	1.9
c.2195C > G	p.S732*	Hetero (2)	3.8
c.593C > T	p.P198L	Homo (1), Hetero (1)	5.8
C.1560–1562 delCCT	Other site	Hetero (1)	1.9
C.2363 T > C	p.H832P	Hetero (1)	1.9
c.1441G > A (p.A481T)	p.A481T	Hetero (1)	1.9
c.1182 + 1G > A	splice site	Hetero (2)	3.8
C.2363 T > C	p.S788 L	Hetero (1)	1.9
c.1832 T > C	p.L611P	Homo (3), Hetero (10)	30.8
c.2359G > A (p.A787T)	p.A787T	Hetero (1)	3.8
c.1349C > T	p.T450 M	Hetero (2)	3.8
c.632C > T	p.P211L	Hetero (2)	3.8
c.1714C > T	p.R572C	Hetero (1)	1.9
c.2180 T > C)	p.L727P	Hetero (2)	3.8
c.1001C > T (p.A334V)	A334V	Hetero (1)	1.9

### In silico analysis novel variants

Our studies revealed four novel variants, which located in *TYR* and *OCA2*, respectively (Table 1 and Fig. 1). The functional sites of the SIFT, PolyPhen2.0 and Mutation Tasters predicted that the novel missense variant of c.2495A > C(p.H832P) likely had deleterious effects, by damaging the function of the OCA2 protein. MutationTaster prediction showed that results displayed that the frameshift mutation of c.561_562insTTATTATGTGTCAAATTATCCCCCA(G190Cfs*12) in TYR, the nonsense mutation of c.2195C > G(p.S732X) and the deletation mutation c.1139-1141delTGG(p.V379I380del) in OCA2 were deleterious. Multiple amino acid sequence alignment of TYR and OCA2 amino acid sequences showed that the novel mutations took place at highly conserved regions (Table 5). According to the ACMG standards and guidelines for the interpretation of sequence variants, these novel mutations is likely pathogenic or pathogenic (Table 5).

**Table 5:** Bioinformatics Analysis of Four Novel Mutations

			Prediction tool					
Gene	Nucleotide change	Amino acid change	PolyPhen-2	SIFT	Mutation Taster	Familial segregation	Vertebrate conservation ^a^	ACMG classification
TYR	c.561_562insTTATTATGTGTCAAATTATCCCCCA	p.G190Cfs*12	NA	NA	Disease causing	Yes	0	pathogenic
OCA2	c.1139-1141delTGG	p.V379I380del	NA	NA	Disease causing	Yes	0	Likelypathogenic
OCA2	c.2495A > C	p.H832P	Probablydamaging	damaging	Disease causing	Yes	0	Likelypathogenic
OCA2	c.2195C > G	p.S732X	NA	NA	Disease causing	Yes	0	pathogenic

Output prediction of each tool—PolyPhen-2 classification: damaging, probably damaging, benign. SIFT classification: damaging, tolerated. MutationTaster classification: disease-causing, polymorphism. Allele frequency: Allele frequencies were checked in dbSNP138, 1000 Genomes Project, HapMap Project, and ExAC database. ^a^Vertebrate conservation: Yes, conserved in more than 80% of all vertebrates aligned; No, conserved in less than 80% of all vertebrates aligned. ACMG classification: pathogenic, likely pathogenic, uncertain significance, likely benign and benign. NA, not available

## Discussion

Oculocutaneous albinism is a recessive hereditary group of diseasesis characterized by reduce or completely absent melanin synthesis despite adequate numbers of structurally normal melanocytes in the skin, hair, and eyes [16]. The common disease-causing genes of OCA are *TYR* and *OCA2* gene. Tyrosinase is a coppercontaining glycoprotein and a oxidase and involved in the formation of pigments, such as melanins and other polyphenolic compounds [11]. The mutations of *TYR* causing the OCA1 are delineated by five key functional sites of the enzyme; two locations copper-binding sites while others are located at the 3′-end of the copper B-binding region near the amino terminus of the protein, and between the CuA and CuB domains [18].

OCA2 is caused by mutations in the *OCA2* gene, resulting in alterationsin the p protein, and which might in turn affect melanin biosynthesis. OCA2 is a transmembrane protein found in the melanosomal membrane. Numerousstudies suggest that this protein could be involved in the transport of tyrosine, the precursor to melanin synthesis within the melanocyte and regulates the pH of melanosome and the melanosome maturation [19–21].

In this study, we identified compound heterozygous and homozygous mutations in *TYR* or *OCA2* in 36 Guangxi Chinese individuals by direct sequencing. The distribution of mutational OCA genes was slightly shifted, OCA2 is the most common type in our oculocutaneous albinism population in Guangxi, China. In a total of 36 OCA patients, 8 were clinically diagnosed with OCA1, 28 were diagnosed with OCA2 (Table 1). Of the 36 molecularly diagnosed patients, 268 carried two mutational alleles and 5 carried one mutational allele. Of the identified patients, we found apparent pathological *TYR* mutations in 22.2% of the patients (8 of 36), OCA2 mutations in 77.8% (28 of 36) (Table 4).

Among patients with OCA1, most of patients genotypically have “tyrosinase-negative” OCA1A and patient 4 have OCA1B, associated with low residual tyrosinase catalytic activity. The mutation c.896G > A(p.R299H) has already been reported [22] which is located in the central portion of tyrosinase and causing the enzyme activity deficient, therefore, the patient was classified as OCA1A. Tomita Yoshioka et al. [23] demonstrated that the frameshift mutation c.929dupC(p.R311Kfs) resulting in a truncated inactive tyrosinase. The heterozygous mutations c.230G > A(p.R77Q) and c.1199G > T(p.W400 L) in the *TYR* gene identified in patient 2 were firstly reported in Japanese and Taiwan patients [24]. The mutation of c.230G > A(p.R77Q) is the major ones in Japanese patients with OCA1A [25]; Chang-Hai Tsai et al. [26] demonstrated that the mutation of c.1199G > T(p.W400 L) might disrupt the second copper binding site of this polypeptide. Therefore, the patients(1–3,5–8) were classified as OCA1A. Patient 4 was compound heterozygous for c.996G > A (p.M332I) and c.1265G > A (p.R422Q) changes in the TYR gene. Grønskov K et al. identified the M332I allele in Denmark for the first time [27], and in 1991, Giebel LB et al. demonstrated the substitution of R422Q results in a tyrosinase polypeptide that is temperature-sensitive [11], the patient was classified as OCA1B. According to the study of Richards S et al., c.346C > T (p.R116X) was a pathegentic mutation, which resulted in premature termination codon downstream [17]. To the best of our knowledge, c.561_562insTTATTATGTGTCAAATTATCCCCCA(G190Cfs*12) was a novel mutation, which was not present in the HGMD Professional Database, dbSNP, or the 1000 Genomes database. The variant caused a frameshift alteration after codon 190 leading to a premature termination codon (PTC) which located at codon 202 and resulting in a inactive tyrosinase .

There, we analyzed the genetic defects underlying OCA2 in 28 albino patients (patients 9–36, Table 1), and identified sixteen known known mutations, three novel mutations. Except the novel mutations, other 16 mutations have been reported [16, 27–35], (https://www.scholarmate.com/S/ivheaf]. Pei-Wen Chiang et al. [24] identified the c.808-3C > G allele in Hispanic for the first time, in their studies, c.808-3C > G would affect the splicing of OCA2 and induce abnormal mRNA splicing. In our study, Seven Patients were inherited this missense substitutions and one patient was homozygous for c.808-3C > G, this is the first time report in Chinese. The c.808-3C > G splicing site mutation represents a significant proportion of the P gene mutations (16.1%) in our oculocutaneous albinism population in Guangxi, China. The site of c.1832 T > C has been reported by Chunyue Miao as a pathogenic mutation [https://www.scholarmate.com/S/ivheaf]. In our studies, there are 14 paitents (18–30, 35) who carries the variant c.1832 T > C in the *OCA2* gene, and the phenotype of the patients were classified as OCA1A or OCA1B. The variant of c.1832 T > C(p.L611P) was occur at the in tracellular regions and it would contribute to the tyrosinase activity in a double heterozygous with other mutational or homozygous state allele in the Chines people. High frequency (30.8%) of mutation c.1832 T > C in our patients, which suggest that L611P may be a common P gene mutation associated with the typical OCA2 phenotype in oculocutaneous albinism population in Guangxi, China. As a common tyrosinase mutation of c.1327G > A(p.V443I) [5, 36], which also have a higher proportion in our albino population. The splice site mutation (c.1182 + 1G > A) is expected to eliminate splicing following exon 11, and causes exons 11–12 skipping, lead to aberrant splicing of the transcript. Aihua Wei et al. [16] identified the c.1182 + 1G > A allele in a China patient for the first time, in their study, they didn’t find the other mutation allele of the patient. In our study, patient 16 and 29 were compound heterozygous for c.1182 + 1G > A and c.1441G > A (p.A481T) or c.1832 T > C (p.L611P) changes in the OCA2 gene. Aihua Wei et al. [16] also identified the c.1560-1562delCCT allele in a Chinese patient for the first time, and the Clinical diagnosis was OCA1B, the variant causing the structure of transmembrane domains. As the study has reported that most missense mutations occur in the loops between the transmembrane domains [37], the mutations of P211L, A334V, V443I, T475P, R455G, P198L, T450 M, A481T, R572C, S788 L, and A787T were close to the interface between the transmembrane domains and the intra- or extracellular regions.

Of the 19 distinct mutations in the *OCA2* gene, three novel mutations have not been presented in dbSNP (http://www.ncbi.nlm.nih.gov/dbvar), the 1000 Genomes Database (http://browser.1000genomes.org/index.html), the HGMD Professional Database (http://www.hgmd.cf.ac.uk/ac/all.php), or the Albinism Datebase (http://www.ifpcs.org/albinism/oca2mut.html). To the best of our knowledge, c.1139-1141delTGG in the *OCA2* gene was a novel mutation, the mutation occur within dinucleotide repeats and may have arisen because of slipped mispairing, and it located at extracellular regions. The mutation of c.2195C > G(p.S732X) change from TAC to TAG which is a novel mutation and located in the structure of transmembrane domains resulted in premature termination codon downstream. The truncated protein lacked transmembrane domains, which might have caused the transport of tyrosine dysfunction and the precursor to melanin synthesis dysfunction and resulted in location of the protein in the nucleus. The c.2495A > C(p.H832P) mutation which located in the structure of transmembrane domains and the hydrophilic histidine becomes hydrophobic proline which may change the transmembrane structure of OCA2, and further affect the tyrosinase activity.

In our study, the clinical phenotypes of 26 OCA2 patients identified showed a wide variety OCA. The Sviderskaya studies [34] shows that the A481T allele had approximately 70% functional activity in melanogenesis compared with that of the wild-type human P cDNA, furthermore, p.481Thr was reported to be an Asian-specific hypopigmentation allele [38]. Jeppe D. Andersen’s date [35] showed that p.481Thr and p.443Ile was sufficient to lower the pigmentation levels in healthy individuals. These findings suggesting that the novel alleles of c.1139-1141delTGG, c.2195C > G(p.S732X) and c.2495A > C(p.H832P),might have no or very low functional activity in melanogenesis. The missense, splicing and deletion mutations destroyed the spatial structure and the integrity of the OCA2 protein, which would delayed the activity of the OCA2 protein different degrees in melanogenesis. Functional studies of the variants is particularly important on the relationships of phenotypes and genotypes of the P gene, and the mechanism for this need futhuer study.

## Conclusion

In summary, we reported thirty six OCA patients and the molecular basis of their disease were identified by PCR-sequencing of all exons of the *TYR* and *OCA2* genes. This study expands the mutational database in humans. It is the first report of the c.1714C > T(p.R572C), c.1139-1141delTGG and c.2195C > G(p.S732X) mutations in the P gene and c.561_562insTTATTATGTGTCAAATTATCCCCCA(G190Cfs*12) in TYR gene. The study shows that OCA2 may be the most common type and the alleles of c.1832 T > C(p.L611P), c.808-3C > G may be the hotspots of OCA2 in oculocutaneous albinism population in Guangxi, China. Molecular genetic testing of *TYR*, *OCA2* is a useful tool for clinical diagnosis and genetic counseling of OCA.

## Additional file

Additional file 1: **Figure S1.** DNA sequencing result from TYR (Patient1–8) and OCA2 (Patient9–36) gene, changes also seen in the father and mother. (DOCX 999 kb)

## Data Availability

All data generated or analysed during this study are included in this published article and its supplementary information files.

## Abbreviations

OCA: Oculocutaneous albinism
*OCA2*: Oculocutaneous albinism II gene
*SLC45A2*: Solute carrier family 45, member 2 gene
*TYR*: Tyrosinase gene
*TYRP1*: Tyrosinase related protein 1 gene
