# Supplementary material for: Genetic analyses of oculocutaneous albinism types 1 and 2 with four novel mutations
Source: BMC Med Genet. 2019 Jun 13;20:106. doi: 10.1186/s12881-019-0842-7 (PMC6567650; doi:10.1186/s12881-019-0842-7)

Additional file 1: Figure S1. DNA sequencing result from TYR (Patient1-8) and OCA2 (Patient9-36) gene, changes also seen in the father and mother


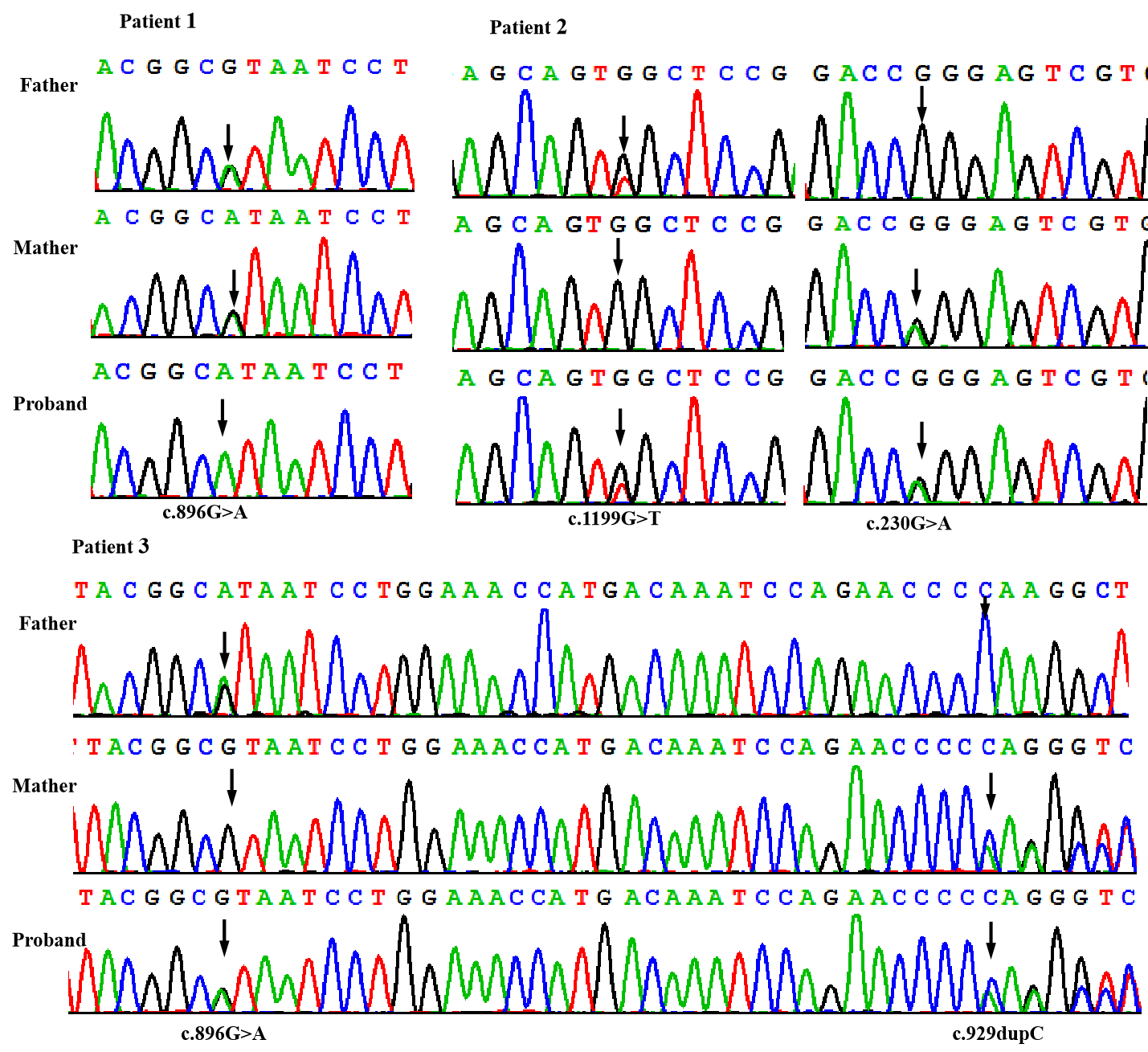


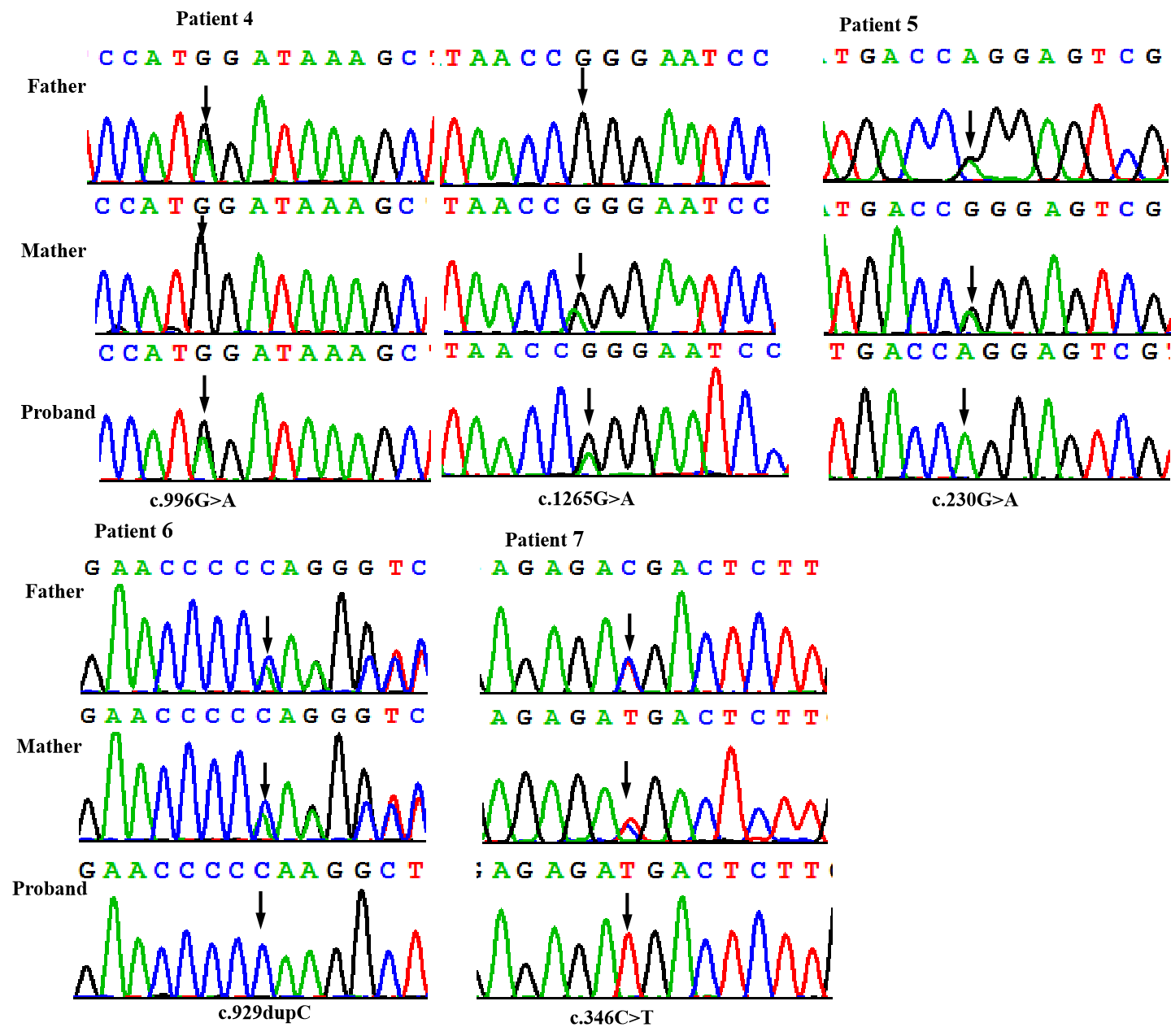

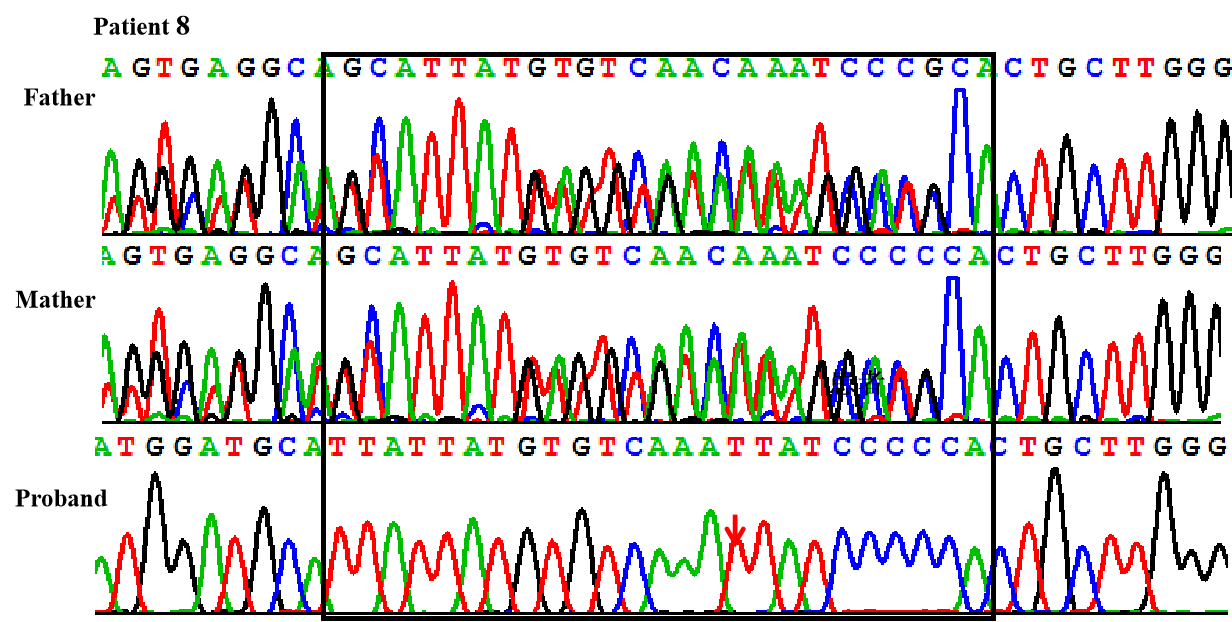


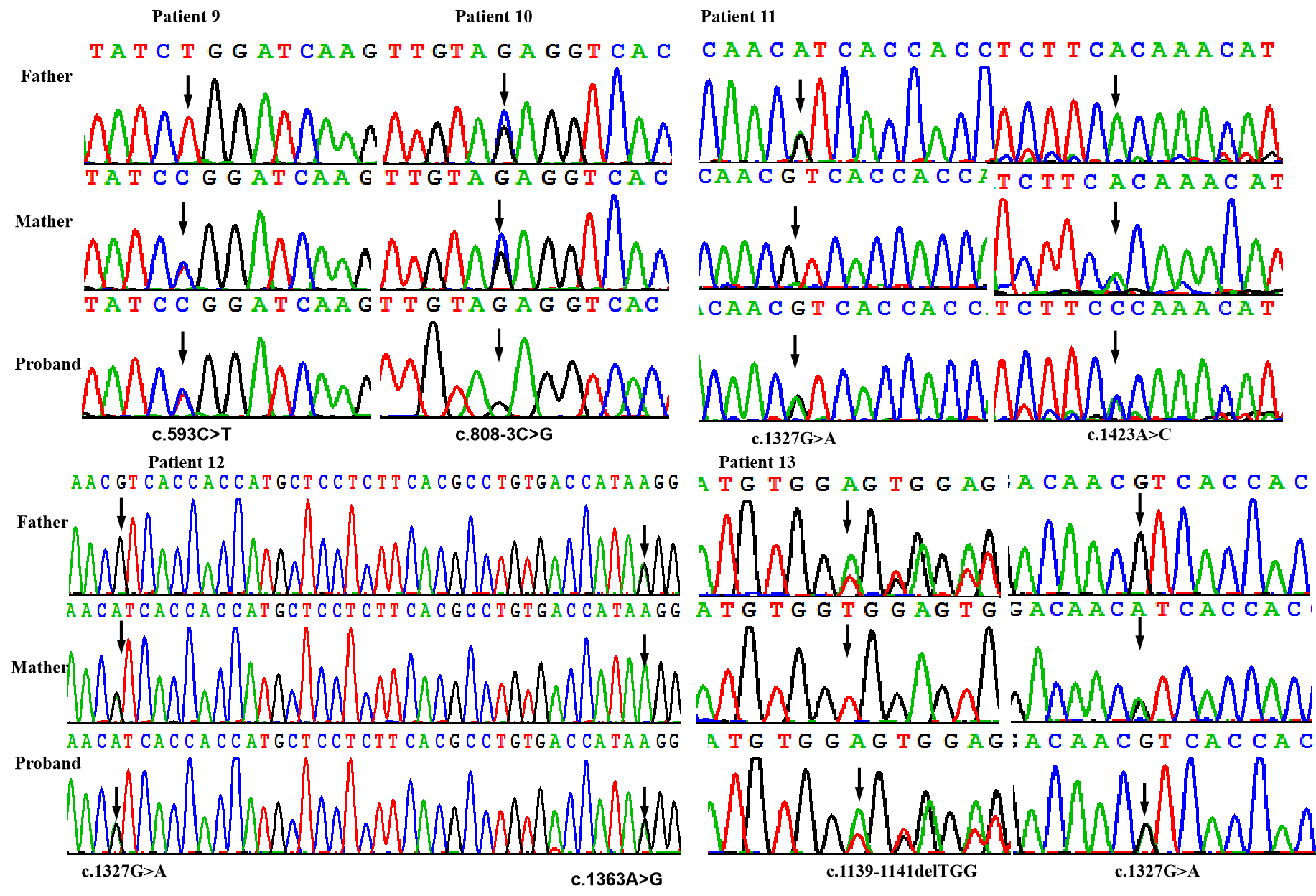

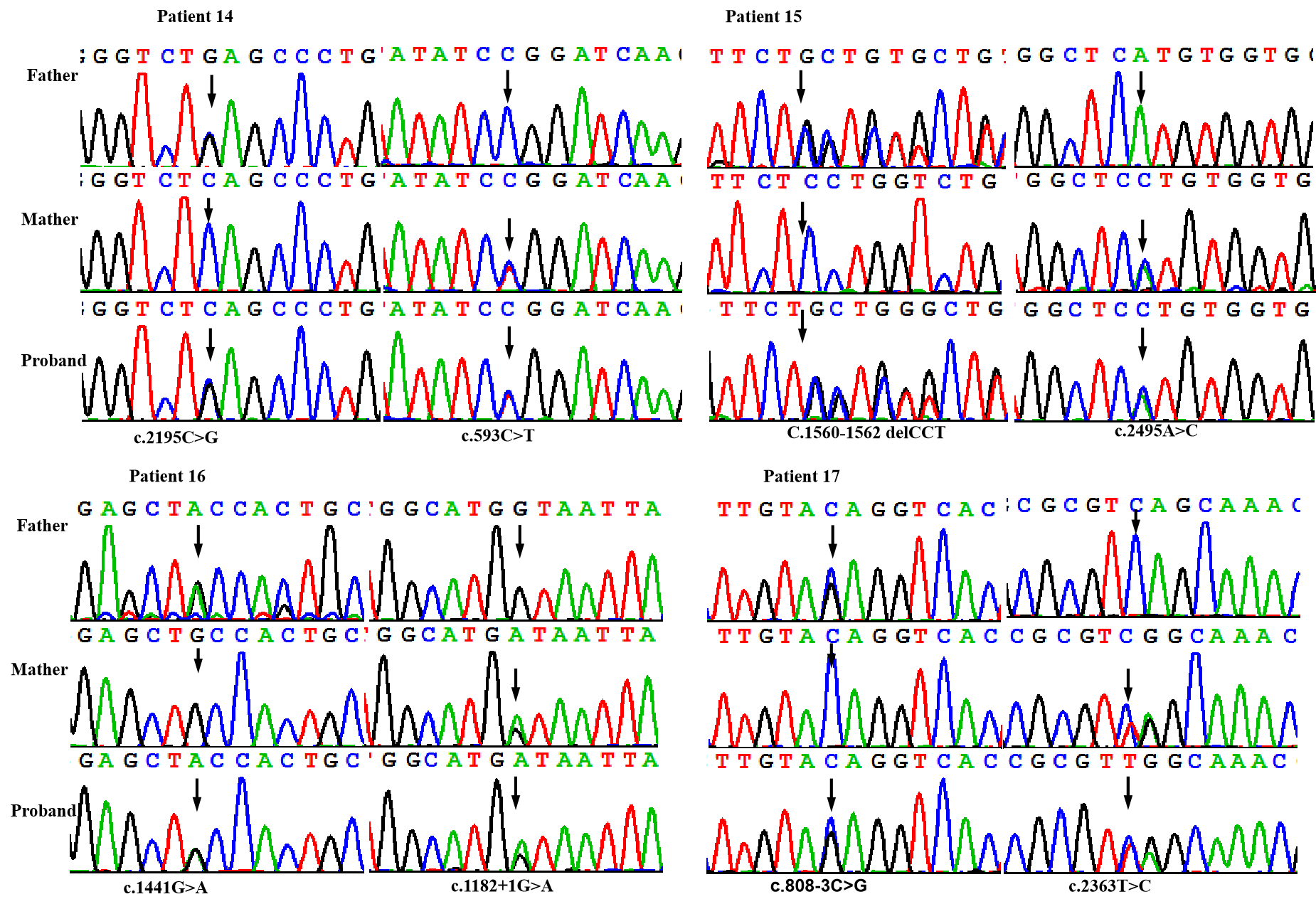


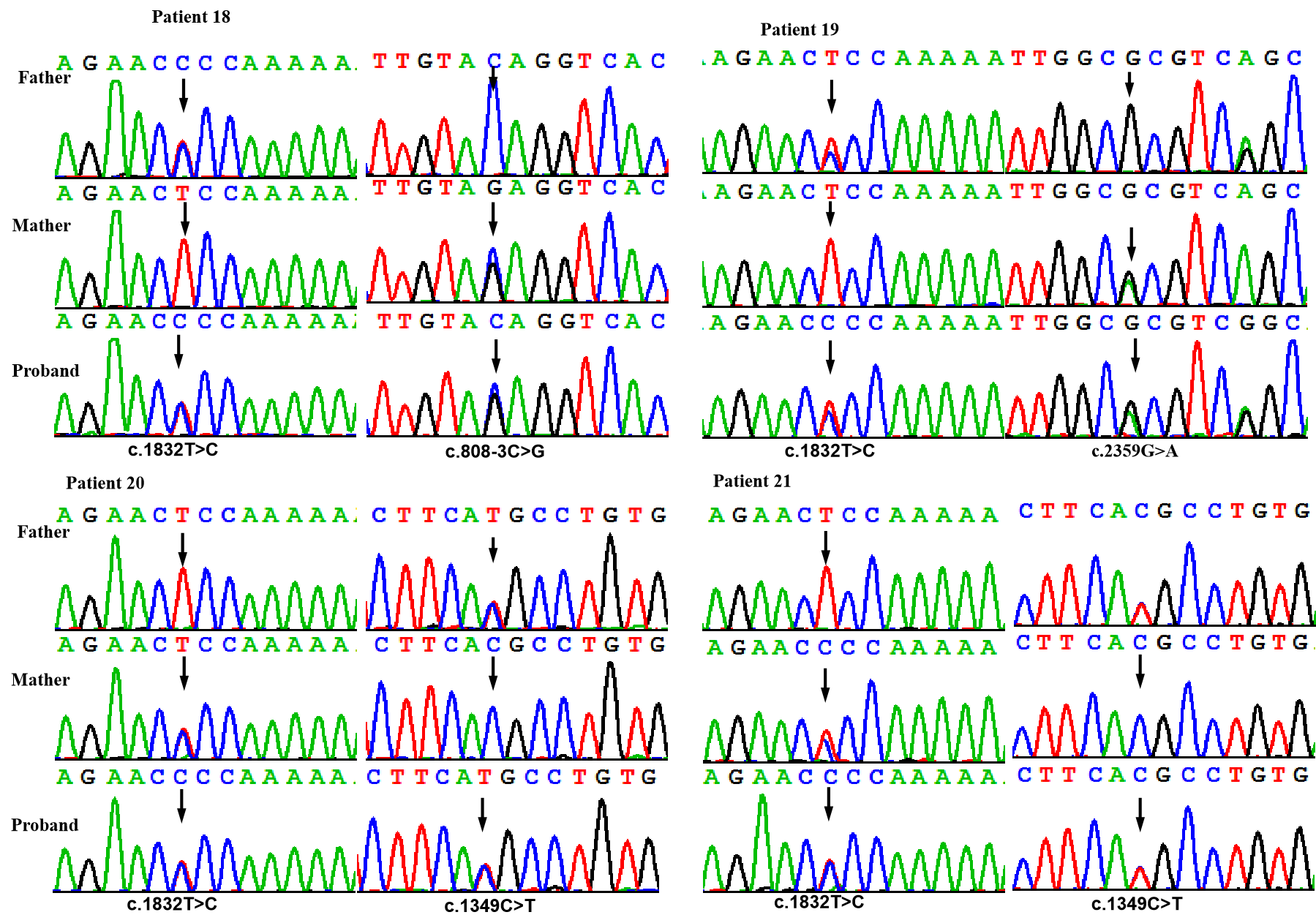


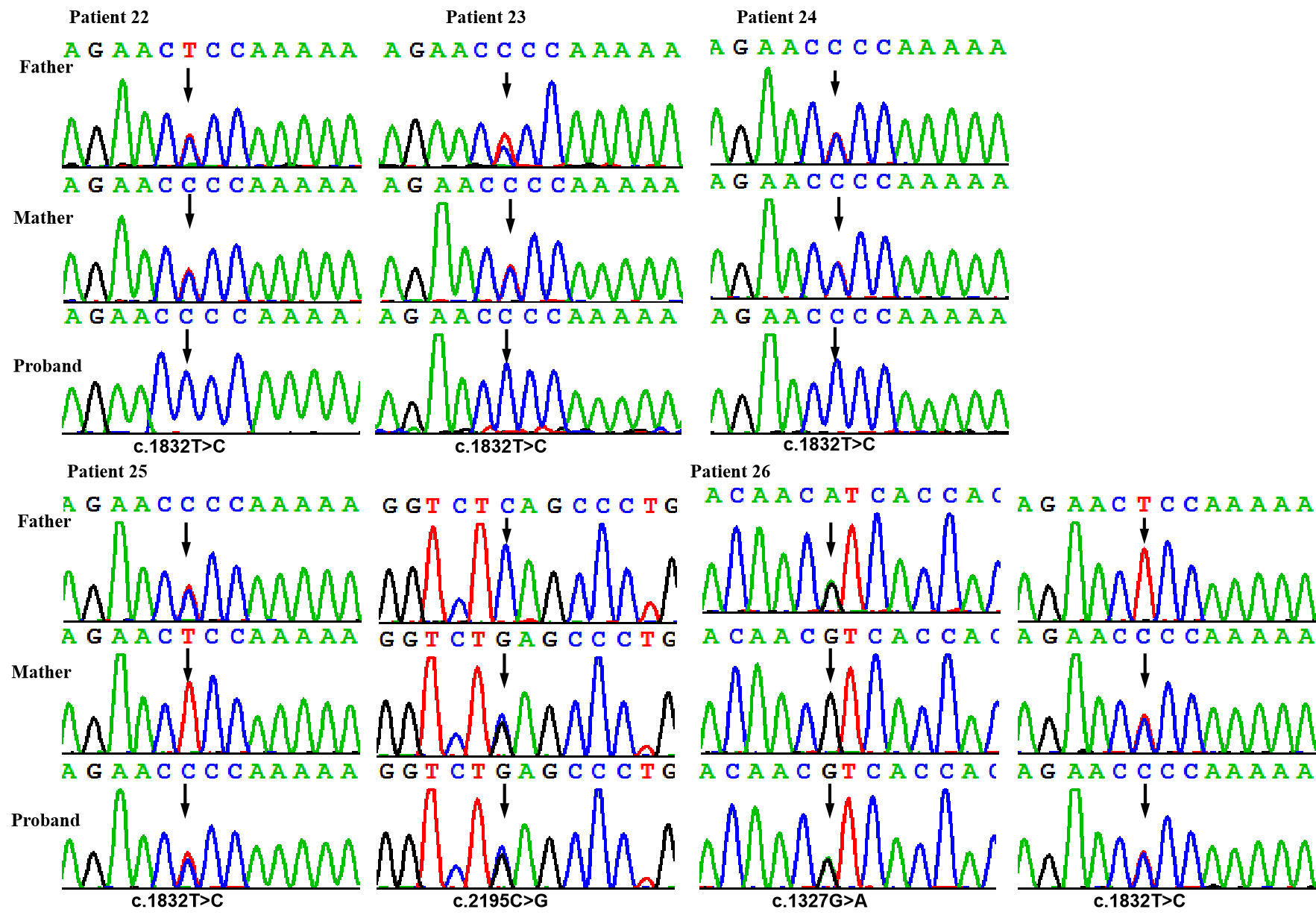


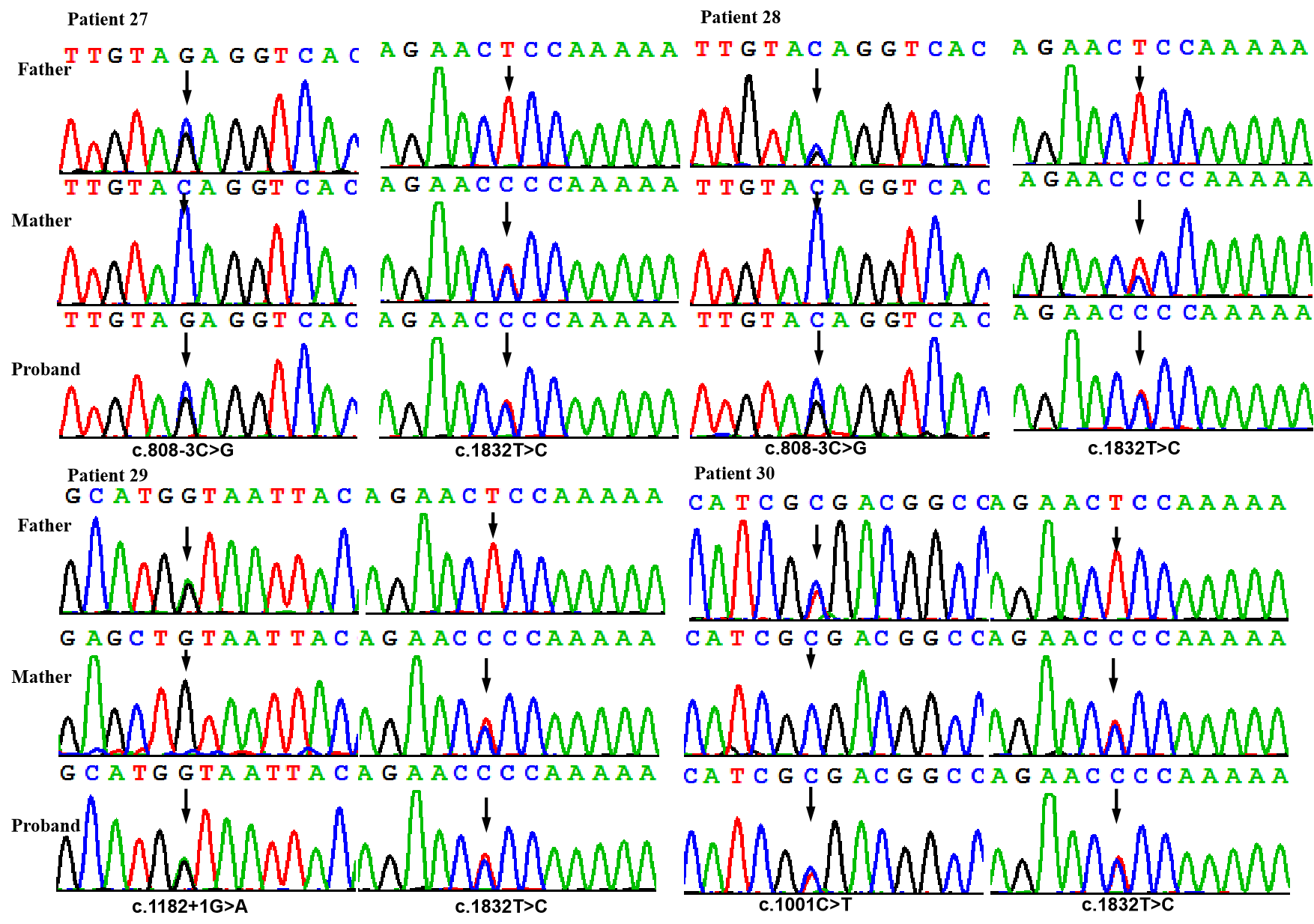


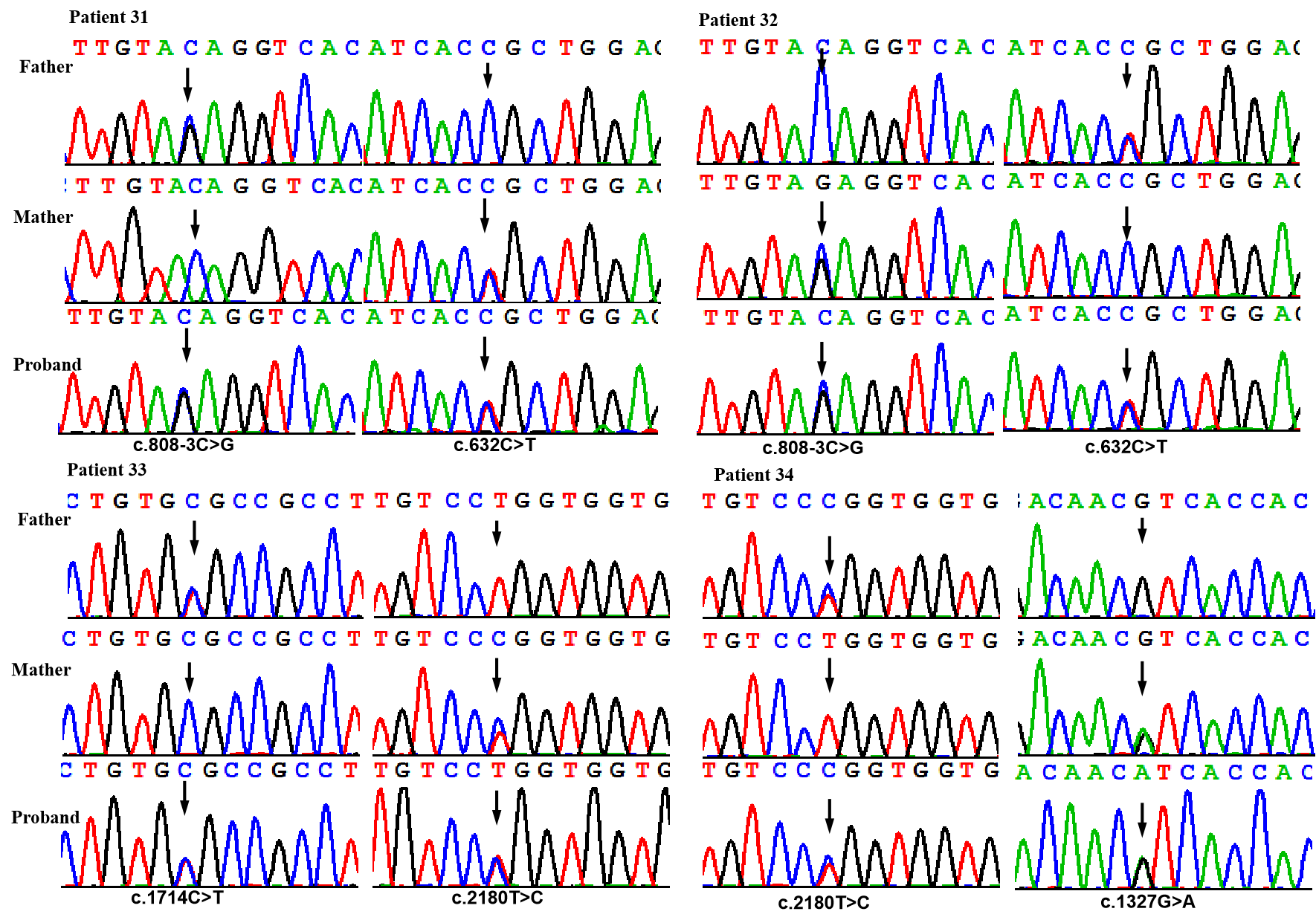


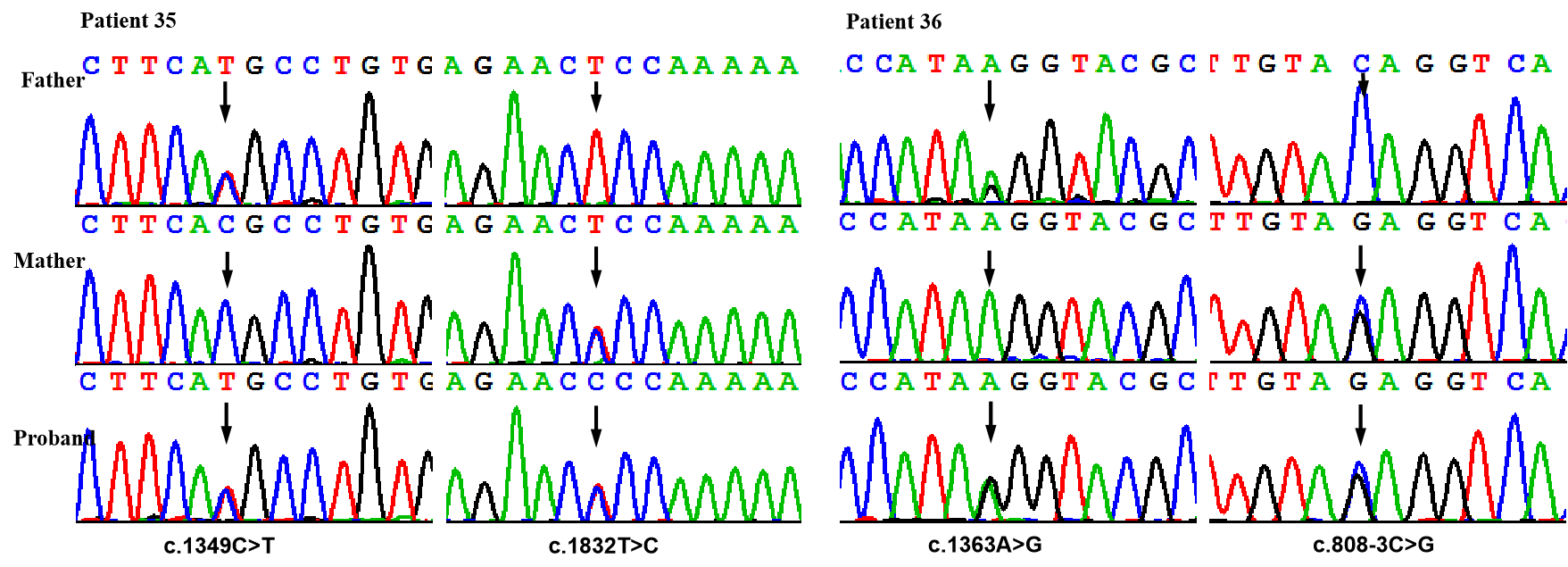

Supplement: Supplementary file 1 — Figure S1. DNA sequencing result from TYR (Patient1–8) and OCA2 (Patient9–36) gene, changes also seen in the father and mother. (DOCX 999 kb) [file 12881_2019_842_MOESM1_ESM.docx]
